# Growth, Yield and Photosynthetic Performance of Winter Wheat as Affected by Co-Application of Nitrogen Fertilizer and Organic Manures

**DOI:** 10.3390/life12071000

**Published:** 2022-07-06

**Authors:** Muhammad Saleem Kubar, Qiang Zhang, Meichen Feng, Chao Wang, Wude Yang, Kashif Ali Kubar, Shagufta Riaz, Hina Gul, Hamz Ali Samoon, Hui Sun, Yongkai Xie, Muhammad Ahsan Asghar

**Affiliations:** 1 College of Resource and Environment, Shanxi Agricultural University, Jinzhong 030801, China; msaleemkubar@yahoo.com (M.S.K.); sunhui@163.com (H.S.); 2 College of Agriculture, Shanxi Agricultural University, Jinzhong 030801, China; fmc10@163.com (M.F.); wcqxx200@126.com (C.W.); 3 Faculty of Agriculture, Lasbela University of Agriculture, Water and Marine Sciences, Uthal 90150, Balochistan, Pakistan; kashifkubar@yahoo.com; 4 Center of Excellence in Marine Biology, Univesity of Karachi, Karachi 75270, Sindh, Pakistan; shagirajput7@gmail.com; 5 University Institute of Biochemistry and Biotechnology, PMAS Arid Agriculture University, Rawalpindi 46300, Punjab, Pakistan; hinagul0y@gmail.com; 6 PARC-Water and Agricultural Waste Management Institute, Tando Jam 70060, Sindh, Pakistan; hamzsamoon@yahoo.com; 7 Institute of Geography Science, Taiyuan Normal University, Jinzhong 030619, China; xieyongki@163.com; 8 Agricultural Institute, Centre for Agricultural Research, ELKH, 2462 Martonvásár, Hungary; ahsanasghar@gmail.com

**Keywords:** wheat cultivars, total chlorophyll content, growth stages, grain yield, Zhongmai 175, Jindong 22

## Abstract

The application of organic manures was found to be beneficial, however, the integrated use of organic manures with chemical nitrogen fertilizers has proven more sustainable in increasing the photosynthetic attributes and grain yield of the winter-wheat crop. A multi-factor split-plot design was adopted, nitrogen and manure fertilizer treatments were set in the sub-plots, including nitrogen-gradient treatment of T1:0 kg N ha^−1^, T2:100 kg N ha^−1^, T3:200 kg N ha^−1^, and T4:300 kg N ha^−1^ (pure nitrogen -fertilizer application) The 25% reduction in nitrogen combined with the manure-fertilizer application includes T5:75 kg N ha^−1^ nitrogen and 25 kg N ha^−1^ manure, T6:150 kg N ha^−1^ nitrogen and 50 kg N ha^−1^ manure, and T7:225 kg N ha^−1^ nitrogen and 75 kg N ha^−1^ manure. The maximum results of the total chlorophyll content and photosynthetic rate were 5.73 mg/g FW and 68.13 m mol m^−2^ s^−1^, observed under T4 in Zhongmai 175, as compared to Jindong 22 at the heading stage. However, the maximum results of intercellular CO_2_ concentration were 1998.47 μmol mol^−1^, observed under T3 in Jindong 22, as compared to Zhongmai 175 at the tillering stage. The maximum results of LAI were 5.35 (cm^2^), observed under T7 in Jindong 22, as compared to Zhongmai 175 at the booting stage. However, the maximum results of Tr and Gs were 6.31 mmol H_2_O m^−2^ s^−1^ and 0.90 H_2_O mol m^−2^ s^−1^, respectively, observed under T7 in Zhongmai 175 as compared to Jindong 22 at the flowering stage. The results revealed that grain yield 8696.93 kg ha^−1^, grains spike^−1^ 51.33 (g), and 1000-grain weight 39.27 (g) were significantly higher, under T3 in Zhongmai 175, as compared to Jindong 22. Moreover, the spike number plot^−1^ of 656.67 m^2^ was significantly higher in Jindong 22, as compared to Zhongmai 175. It was concluded from the study that the combined application of nitrogen and manure fertilizers in winter wheat is significant for enhancing seed at the jointing and flowering stages. For increased grain yield and higher economic return, Zhongmai 175 outperformed the other cultivars examined. This research brings awareness toward the nitrogen-fertilizer-management approach established for farmers’ practice, which might be observed as an instruction to increase agricultural management for the winter-wheat-growth season.

## 1. Introduction

Population load, poverty, agricultural expansion and intensification, and development of infrastructure have been suggested as major threats to soil structure and biodiversity [1,2,3]. Due to the above-mentioned disturbances, land-use systems have been affected adversely in terms of microbial activities, nutrient cycling, and reduced the soil productivity [4,5,6]. To cope with this situation, options are often used to make the soil more productive and sustainable. Cereals are an essential crop all over the world, since they originate the key protein and energy supply for most countries [7]. Winter wheat is a usually a cultivated crop in northern China. The compatibility of techniques to increase wheat-grain yield is progressively essential for grain production and food security in China [8]. Nitrogen is a vital nutrient for crop growth and grain formation. Farming administrators can control N organizations at the proper rates and right times based on the crop’s N supplies [9]. Late season or splitting applications of N fertilizer are common methods to increase grain yield and nitrogen-use efficiency. Plant biomass was superior under splitting N applications (applied at the returning green stage and jointing stage) lower than below total N applications at the tillering stage [10]. While splitting fertilizer application needs extra labor, the present fertilization technique is usually used on crops below conventional farming practices [11]. After all, this fertilization technique is currently unsuitable in China; for example, the worker community is aging and laborers’ absence for crop-production activities is becoming progressively terrible [12]. Therefore, balancing the profits obtained from fertilizers with the related environmental problems is ultimately required.

Persistent use of chemical composts causes health and environmental issues, such as ground and surface-water poisoning. Hence, reducing the quantity of synthetic N fertilizers will cause nitrogen insufficiency in agricultural soils, which would be a primary concern in agricultural practices. Expending organic materials could be one of the options for reducing the need for synthetic fertilizers [9,11]. Organic matter is widely acknowledged as having a significant function in maintaining high soil fertility. Various studies have confirmed the favorable impacts of manure fertilizers on soil fertility and crop-yield quality [13,14,15,16]. Similarly, a few studies have demonstrated that manure-fertilizer technologies are more secure for plants and the atmosphere than chemical products. Inadequate manure-fertilizer application can pollute surface and ground water, might bring a plant nutrient toxicity and deficiency, or cause salt damage. Seed inoculation of wheat cultivars with manure fertilizer revealed a significantly improved grain yield and productive growth [17].

Applying chemical fertilizers is a good technique for improving the deficiency of nutritional components, though they not only enhance the amount of production but also are sometimes insufficient to meet farmers’ demands [9]. While chemical fertilizers are very dynamic in improving grain yield, they may decline the structure of the soil and pollute the surface water. Moreover, due to the energy crisis, fertilizers are expensive and unavailable to many farmers, especially in developing countries like China and Bangladesh [11,15]. In this situation, manure has become popular with farmers as a cheaper source of plant-based food. It increases soil fertility when it decomposes. At the same time, increasing soil-organic-matter content can also improve its water-holding capacity, aeration, and colloidal complexes, thereby improving its ability to store nutrients [16].

In order to solve this problem, fertilization has become an important method to enrich soil fertility and accumulate water to meet plant-growth requirements and increase crop yield on the Loess Plateau [18]. However, local farmers’ neglect and excessive use of fertilizers has resulted in a series of environmental problems, including eutrophication, ground-water pollution, and greenhouse-gas emissions [19]. Rather than simply boosting crop yield, the rapid expansion of agriculture has resulted in an improvement in soil fertility and environmental protection, allowing dry-land agriculture to perform profitably. Various studies have described that the combined application of nitrogen with manure fertilizer plays a significant role in increasing soil nutrients, crop yields, and chemical-fertilizer- and nitrogen-use efficiency [20,21,22]. However, an uncooperative mixture (percentage) of manure and nitrogen rates did not result in high crop yields and nitrogen-use efficiencies, but instead produced a huge quantity of soil nitrate N residue accretion, which transferred down to deeper soil [23]. Furthermore, it pollutes the groundwater beneath the surface. Therefore, the current research aimed to: (1) observe the combined influence of nitrogen with manure fertilizer on the grain yield, photosynthetic parameters, LAI, and chlorophyll content of winter wheat; (2) explore the optimal combination of manure and N fertilizer for two cultivars of winter-wheat production and environment protection; and (3) examine the responses of gas exchange and chlorophyll content to various manure- and nitrogen-application conditions, and the relationship of these traits with the grain yield of winter wheat. The outcomes of this research will provide a hypothetical basis for dry-land winter-wheat production and soil-fertility buildup.

## 2. Materials and Methods 

### 2.1. Experimental Locations 

Field trials were accompanied during 2020 to 2021 on Taigu experimental farming station of Shanxi Agricultural University, (37°25′ N, 112°33′ E), Shanxi province, China. The research area, at Taigu base, has temperate climate with annual temperature of 12 °C and annual rainfall of 442 mm, with possible evapotranspiration of 1840.2 mm and 2672 period of sunlight. The investigational zone is a mountainous arid field with a semiarid weather characteristic of the northeast Loess Plateau, where 60% to 70% rainfall followed in the seasonal months through the fallow season (July to August). The surface soil of established field has a 7.7 mg kg^−1^ soil organic matter, 7.2 pH, and 51.12, 19.34, and 243.26 mg kg^−1^ of available nitrogen, phosphorus, and potassium, respectively. 

### 2.2. Treatments Detail

The experimental field is located in Shenfeng base of Shanxi Agricultural University, Taigu, Shanxi province, China (37°26′ N, 112°35′ E). The plot area was 20 m^2^ (4 × 5). Varieties tested: Jindong 22 (crude protein content 17.4%) and Zhongmai 175 (crude protein content 13.5%). Multi-factor split-plot design was adopted in the experiment; the main plot was different wheat varieties, and the secondary plot was fertilizer treatments (pure N). The different wheat varieties in the main area were Jindong 22 (South) and Zhongmai 175 (north), referred to as Jindong 22 and Zhongmai 175. Seven fertilizer treatments (pure N) were set in the sub-area, including nitrogen treatment of 0 kg/hm, 100 kg/hm, 200 kg/hm, and 300 kg/hm (pure nitrogen-fertilizer application), and 25% reduction in nitrogen combined with organic (chicken)-manure-fertilizer application (1.5% N content). Mark the total nitrogen content and 100 kg/hm (75% nitrogen + 25% chicken manure), 200 kg/hm (150% nitrogen + 50% chicken manure), and 300 kg/hm (225% nitrogen + 75% chicken manure) as equal, namely organic fertilizer:chemical fertilizer (pure N content) = 1:3, base ratio is 5:5, manure fertilizer was used as base fertilizer. Each treatment was repeated three times. Urea (46.1% N content), phosphate (16% P_2_O_5_ content) and potassium (50% K_2_O content) were applied at 120 kg/hm as base fertilizer. Other farming activities are the same as local wheat-yield management. All the other agronomic practices, such as irrigation, insecticide/pesticide application, and weed control were uniformly and properly farmed according to plant growth stage and requirements, following Shanxi province’s traditional agricultural practices.

### 2.3. Leaf Area Index

Leaf-area index (LAI) of winter wheat below N ratios and timings, in different treatments, was determined at jointing, flowering, and grain-filling stages in both years. For this purpose, 20 cm winter-wheat plants from the main rows of all the sub-blocks were detrimentally harvested at least one rhythm apart from the previous sampling. To adjust the leaf area of winter wheat plants, we dignified the high leaf distance with a ruler, formerly leaf area was measured by multiplying the leaf length and width with a constant of 0.75 [17].

### 2.4. Photosynthesis Parameters

As described by [10,14] the (Pn) photosynthesis rate, (Gs) stomatal conductance, (Ci) Intercellular CO_2_ absorption, and (Tr) transpiration rate of winter-wheat crop under nitrogen levels, nitrogen ratios, and N timings were observed with the use of Li-6400 convenient photosynthesis method (LI-COR Inc., Lincoln, NE, USA), prepared with an LED leaf chamber. From each treatment, two fully prolonged flag leaves plot^−1^ from each treatment of the winter-wheat crop were selected at jointing, flowering, and grain-filling stages to determine the photosynthesis traits. All the dimensions were accompanied from 10:00 to 11:10 on a clear sunny day with a 400 mol mol^−1^ of CO_2_ concentration

### 2.5. Spectrophotometer Method

Synchronized with the photosynthesis measurements, collect two leaves of winter wheat, immediately place them into the sealed bag, for each processing collection. Remove the leaves from the middle of the leaves in the lab and cut them into small pieces of less than 2 mm in width. Accurately weigh 0.08 g into the 25 mL volumetric flask, with 80% acetone volume, sealed under dark conditions for 24 h. The absorbance at 470 nm, 663 nm, 645 nm, and 652 nm was determined by Shimadzu UV-1800 UV-Vis spectrophotometer. 

### 2.6. Yield Components

The yield traits were the number of grains spike^−1^, number of spike plot^−1^, and the 1000-grain weight for seeds, and the winter-wheat yield was measured by harvesting the winter wheat from the central rows of individual plot. At maturity, 1 m^2^ winter wheat plants from individual treatment of all replications were randomly selected. These plants were harvested by cutting at the soil level with sickle. The ear heads were disconnected from straw, retained in separate paper bags, oven dried for 24 h at 78 °C, and each sample was threshed manually. The observations were documented by the following parameters: The number of spikes per plot for each spike of the casually selected plants were calculated at the crop ripeness, and means were driven out. The grains spike^−1^ for each randomly selected plant were calculated during the crop maturation, and means were computed. In total, 1000 grains from each plot were randomly collected and weighed to compute the seed index in grams (g). The grains received from each plot were balanced, and on the basis of grain yield per plot, grain yield per hectare was calculated in kilograms (kg). Grain yield (Gy14% moisture content), and dry-matter weight were measured as described by [21].

$$\mathrm{Grain}\;\mathrm{yield}\left(\mathrm{kg}/\mathrm{ha}\right)=\frac{\mathrm{Spike}\;\mathrm{number}\;\mathrm{plot}-1\times \;\mathrm{grain}\;\mathrm{number}\;\mathrm{spike}-1}{1000\;\mathrm{grains}\;\mathrm{weight}}$$

### 2.7. Statistical Analysis

The data presented in this study were the average of three replications. All data were analyzed using ANOVA for a randomized complete-block design. The significant of each source was determined by F-test. Duncan’s Multiple Range Test (DMRT), significant difference was used as a post hoc mean-separation test (*p* < 0.05) using SAS 9.3 (SAS Institute, Cary, NC, USA). Treatments were compared on the basis of significant differences with least significance difference (LSD *p* < 0.05). Shapiro–Wilk test was performed to assess the normality of variance before assessing with ANOVA. Microsoft Excel 2013 (Redmond, WA, USA) was used in data calculation, while Origin 8.5 (Farmington, ME, USA) was used in graphical representation. All the statistical analysis was performed using SPSS version 19.0 (Chicago, IL, USA) and SAS version 9.3 (Cary, NC, USA).

## 3. Results

### 3.1. Yield and Components

Effects of nitrogen and nitrogen and manure treatments on the grain-yield components of winter wheat are presented in (Table 1). In the whole growing season, the highest yield was 8696.93 kg ha^−1^ and 8248.19 kg ha^−1^ under T3 (Pure N) treatments in both cultivars Jindong 22 and Zhongmai 175, respectively; however, Zhongmai 175 was higher than Jindong 22. In comparison with the control, the yield was increased 44.19% and 53.27%, respectively. The lowest results were the 6644.86 kg ha^−1^ recorded at the T2 treatment under Zhongmai 175, and the 6662.93 kg ha^−1^ recorded at T7 (organic) under Jindong 22, as compared to the control. During the time period of winter wheat, the highest number of grains spike^−1^ were the 656.67 m^2^ and 462.67 m^2^ that appeared in T3 (Pure N), which was increased by 23.43% and 1.98% respectively, compared with the control treatment. Again, Jindong 22 was higher than Zhongmai 175. The lowest results were the 552.67 m^2^ recorded at the T5 treatment (organic) under Jindong 22, and the 356.67 m^2^ recorded at T4 (nitrogen) under Zhongmai 175, as compared to the control. 

The results revealed that the maximum 1000-grain weight of winter wheat were the 38.12 g and 39.27 g that appeared in the T3 (Pure N) treatment in both cultivars However, Zhongmai 175 was greater than Jindong 22, which increased by 20.56% and 25.85% respectively, compared with the control treatment. The lowest results, of 33.40 g and 35.30 g, were observed under the T4 (Pure N) and T6 (nitrogen and manure) treatments, respectively, and the T2 (Pure N) and T6 (nitrogen and manure) treatments, as compared to the control. In our study, the maximum number of grains spike^−1^ of winter wheat were the 42.22 g and 51.33 g recorded in the T3 treatment, but in both cultivars Zhongmai 175 was higher than Jindong 22, as compared to the control, which increased by 42.30% and 47.59%, respectively, compared with the control treatments. However, the minimum results were the 31.11 g and 38.78 g observed under the T2 (Pure N) treatments in both cultivars, as compared to the control.

### 3.2. Leaf-Area Index (LAI) (cm^2^)

**Figure 1:**
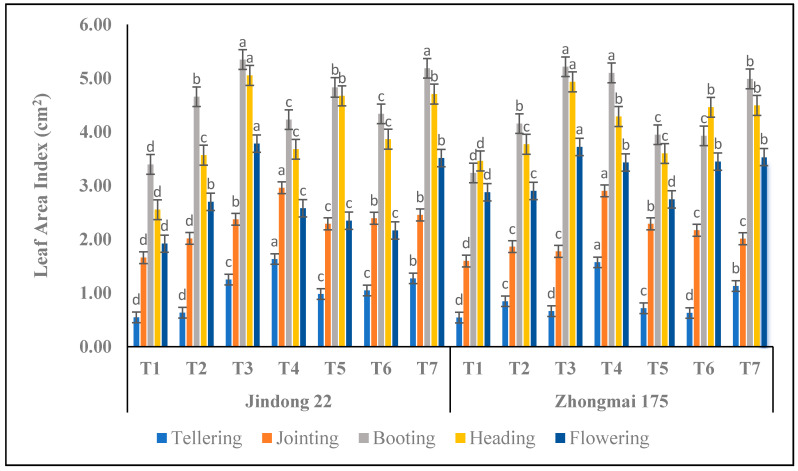
**Leaf-area index of wheat crop as affected by the integrative effects of the nitrogen and manure application.** T1 (control plot), T2 (nitrogen 100 kg^−1^ ha), T3 (N 200 kg^−1^ ha), T4 (N 300 kg^−1^ ha), T5 100 kg^−1^ ha (nitrogen 75% + manure 25%), T6 200 kg^−1^ ha (nitrogen 50% + manure 50%), and T7 300 kg^−1^ ha (nitrogen 25% + manure 75%). Mean values in separate columns followed by similar letters are not significantly different at *p* < 0.05. The values are the mean ± SE (*n* = 3).

Leaf-area index of the winter-wheat crop revealed significant differences (*p* < 0.05), since the tillering to flowering period was below the progressive nitrogen rate, with the cultivars are shown in Figure 1. LAI increased with the increase in the nitrogen-application amount. In the winter-wheat growing period, the maximum value of LAI was mainly concentrated in the tillering and jointing periods under T4 in both cultivars, at the booting, heading, and flowering stages under the T7 (inorganic) treatment in both cultivars, as compared to the control. Overall, LAI increased significantly under the T7 (inorganic) treatment compared with the control at the booting stages in both the Jindong 22 and Zhongmai 175 cultivars. In our experiment, in terms of Jindong 22, LAI in the T7 treatment was greater than in the control plots in all growth stages of the winter-wheat crop. As far as Zhongmai 175 is concerned, LAI under T7 treatment was significantly higher than under the control plot at the booting stage. Overall, the maximum LAI were the 5.35 (cm^2^) and 5.21 (cm^2^) recorded under T7 at the booting stage, as compared to the control, but Jindong 22 was higher than Zhongmai 175. However, the lowest results were 0.63 and 0.63 recorded under T2 and T6 (inorganic) at the tillering stage in both cultivars.

### 3.3. Chlorophyll Content (CC) (mg/g FW)

**Figure 2:**
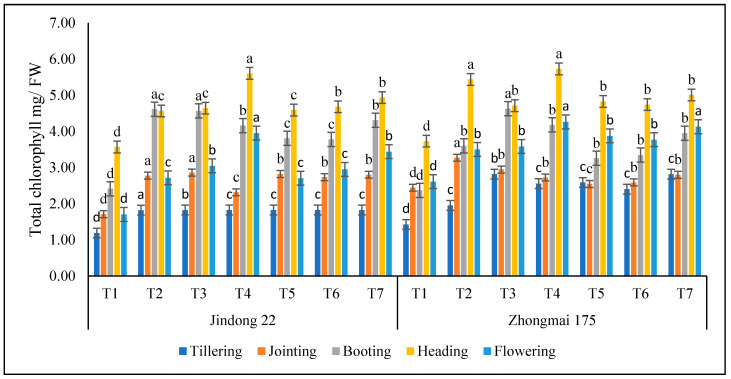
Total chlorophyll content as affected by the integrative effects of the nitrogen and manure application of winter wheat. T1 (control plot), T2 (nitrogen 100 kg^−1^ ha), T3 (N 200 kg^−1^ ha), T4 (N 300 kg^−1^ ha), T5 100 kg^−1^ ha (nitrogen 75% + manure 25%), T6 200 kg^−1^ ha (nitrogen 50% + manure 50%), and T7 300 kg^−1^ ha (nitrogen 25% + manure 75%). Mean values in separate columns followed by similar letters are not significantly different at *p* < 0.05. The values are the mean ± SE (*n* = 3).

The study observed that the total chlorophyll content of flag leaves in the winter-wheat growth period may reflect the supply of soil nitrogen. The total chlorophyll content values of winter-wheat leaves increased with the nitrogen-application rate during the growth period, as shown in Figure 2. In terms of chlorophyll content, Jindong 22 and Zhongmai 175 reached the highest chlorophyll content of 5.60 mg/g FW and 5.73 mg/g FW, respectively, under the T4 treatments at the heading stage, in the whole growing season of the winter-wheat crop as compared to the control. The tillering, jointing, and booting stages under T3 were higher than the control, but the heading and flowering stages under T4 (Pure N) were higher than the control in both cultivars. Among which, the chlorophyll content in T4 increased by 30.01% and 53.58% as compared to the control plot. However, the minimum results were 1.82 and 1.95, recorded for Jindong 22 and Zhongmai 175 under T2 at the tillering stage.

### 3.4. Photosynthetic Rate (μmol m^−2^ s ^−1^)

**Figure 3:**
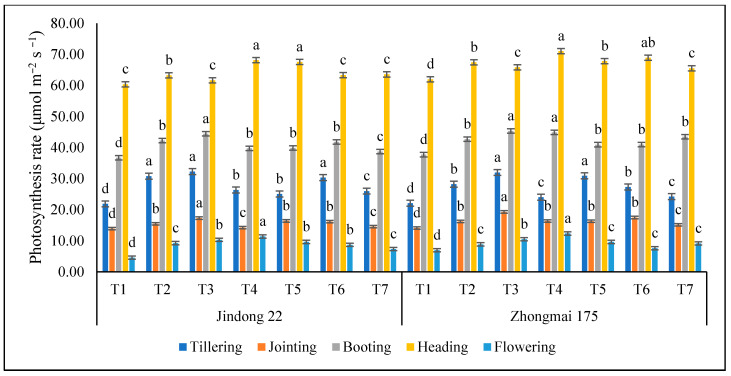
Photosynthesis rate as affected by the integrative effects of the nitrogen and manure application of winter wheat. T1 (control plot), T2 (nitrogen 100 kg^−1^ ha), T3 (N 200 kg^−1^ ha), T4 (N 300 kg^−1^ ha), T5 100 kg^−1^ ha (nitrogen 75% + manure 25%), T6 200 kg^−1^ ha (nitrogen 50% and manure 50%), and T7 300 kg^−1^ ha (nitrogen 25% and manure 75%). Mean values in separate columns followed by similar letters are not significantly different at *p* < 0.05. The values are the mean ± SE (*n* = 3).

The photosynthetic-light-response curve effectively reflects the photosynthetic characteristics of winter-wheat plants and nitrogen. The trend in photosynthesis light response curve of Jindong 22 and Zhongmai 175 in different growth stages are summarized in Figure 3. In a certain range, the PN increased with the increase in nitrogen application. In general, the PN of winter wheat at the heading stages were greater than those at the tillering and flowering stage. As for Jindong 22, the PN reached the maximum values of 68.13 mmol m^−2^ s^−1^ under the T4 (inorganic) treatment at the heading stage in the whole growing season, which were 12.87% greater than the control plot. As for Zhongmai 175, the PN at the heading stage under T4 (inorganic) in the winter-wheat growing season reached the maximum value of 70.99 mmol m^−2^ s^−1^ recorded for both cultivars, but Zhongmai 175 is higher than Jindong 22, as compared to the control, which were up to 14.55% higher than the control plot. Minimum results were 11.36 and 12.33 recorded under T4 (inorganic) at the flowering stage, as compared to the control and all growth stages.

### 3.5. Stomatal Conductance (GS) (mol H_2_O m^−2^ s^−1^)

**Figure 4:**
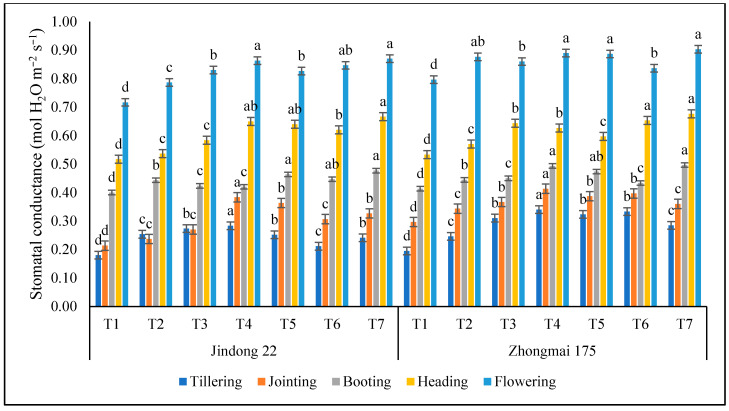
Stomatal conductance as affected by the integrative effects of the nitrogen and manure application of winter wheat. T1 (control plot), T2 (nitrogen 100 kg^−1^ ha), T3 (N 200 kg^−1^ ha), T4 (N 300 kg^−1^ ha), T5 100 kg^−1^ ha (nitrogen 75% and manure 25%), T6 200 kg^−1^ ha (nitrogen 50% and manure 50%), and T7 300 kg^−1^ ha (nitrogen 25% and manure 75%). Mean values in separate columns followed by similar letters are not significantly different at *p* < 0.05. The values are the mean ± SE (*n* = 3).

In terms of stomatal conductance (GS), Jindong 22 and Zhongmai 175 reached the highest GS at 0.87 H_2_O mol m^−2^ s^−1^ and 0.90 H_2_O mol m^−2^ s^−1^ under the T7 (Inorganic) treatment at the flowering stages, respectively, as summarized in Figure 4. Among th both cultivars, Zhongmai 175 was higher than Jindong 22, compared to the control. Among which, the GS in T4 and T7 increased by 82.54% and 28.21%, respectively, at the jointing and heading stages, as compared to the control, under Jindong 22, while the GS in T4 and T7 increased by 78.95% and 27.67%, respectively, under Zhongmai 175 at the tillering and heading stages. At the tillering and jointing stages, GS under T4 (pure N) was higher than the control and at the booting, heading, and flowering stage, while the GS under T7 (inorganic) was higher than the control. And the lowest, results were 0.21 and 0.25 observed under T6 (organic) and T2 (pure N), compared to the control plot.

### 3.6. Intercellular CO_2_ Concentration (CI) (μmol CO_2_ m^−2^ s^−1^)

**Figure 5:**
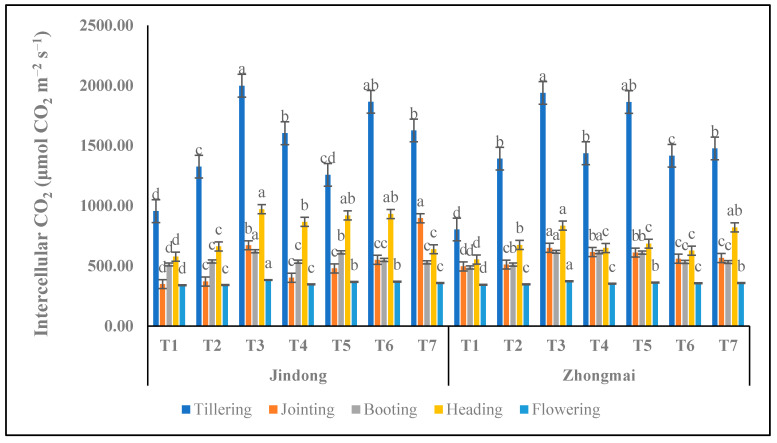
Intercellular CO_2_ concentration as affected by the integrative effects of the nitrogen and manure application of winter wheat. T1 (control plot), T2 (nitrogen 100 kg^−1^ ha), T3 (N 200 kg^−1^ ha), T4 (N 300 kg^−1^ ha), T5 100 kg^−1^ ha (nitrogen 75% and manure 25%), T6 200 kg^−1^ ha (nitrogen 50% and manure 50%), and T7 300 kg^−1^ ha (nitrogen 25% and manure 75%). Mean values in separate columns followed by similar letters are not significantly different at *p* < 0.05. The values are the mean ± SE (*n* = 3).

Nitrogen affected the intercellular CO_2_ concentration (CI) of winter wheat (Figure 5). CI increased with the increase in nitrogen-application amount. In the winter-wheat growing period, the maximum values were 1998.47 μmol mol^−1^ and 1904.85 μmol mol^−1^ of CI, which were mainly concentrated in the tillering stages. CI increased significantly under the T3 treatment compared with the control treatment at all the growth stages in both cultivars, while Jindong 22 performed better than Zhongmai 175, which increased by 109.27% and 137.23%. The minimum results were 340.14 and 346.20, recorded under T2 compared to the control.

### 3.7. Transpiration Rate (Tr) (mmol H_2_O m^−2^ s^−1^)

**Figure 6:**
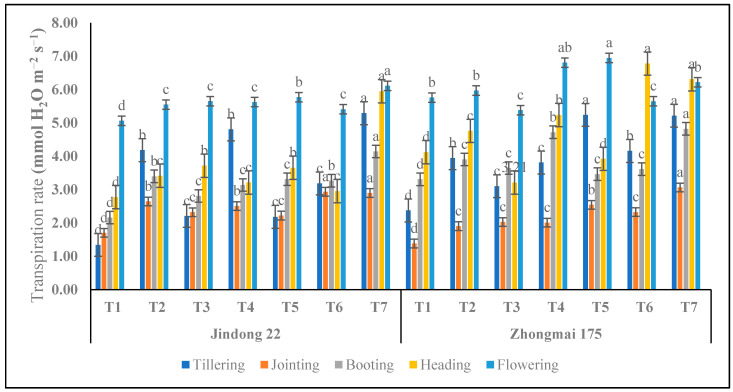
Transpiration rate as affected by the integrative effects of the nitrogen and manure application of winter wheat. T1 (control plot), T2 (nitrogen 100 kg^−1^ ha), T3 (N 200 kg^−1^ ha), T4 (N 300 kg^−1^ ha), T5 100 kg^−1^ ha (nitrogen 75% and manure 25%), T6 200 kg^−1^ ha (nitrogen 50% and manure 50%), and T7 300 kg^−1^ ha (nitrogen 25% and manure 75%). Mean values in separate columns followed by similar letters are not significantly different at *p* < 0.05. The values are the mean ± SE (*n* = 3).

In terms of transpiration rate (Tr), Jindong 22 and Zhongmai 175 reached the highest Tr, at 5.95 to 6.11 mmol H_2_O m^−2^ s^−1^ and 6.31 to 6.22 mmol H_2_O m^−2^ s^−1^ under the T7 (inorganic) treatments at the tillering, heading, and flowering stages, respectively, in the whole growing season of the winter-wheat crop, as summarized in Figure 6. Among which, the Tr under the T7 treatment increased by 113.96% to 294.79% for Jindong 22, as compared to the control plot, and for Zhongmai 175 it increased by 53.12% to 119.14%, as compared to the control plot. Moreover, Zhongmai 175 was higher than Jindong 22 compared to the control under the T7 treatment at the heading and flowering stages, respectively. However, the minimum results of 2.22 and 2.01 were recorded for Jindong 22 and Zhongmai 175 under T4 and T5 (inorganic) at the jointing stages.

### 3.8. Relationship between Grain Yield and the Other Studied Parameters

**Figure 7:**
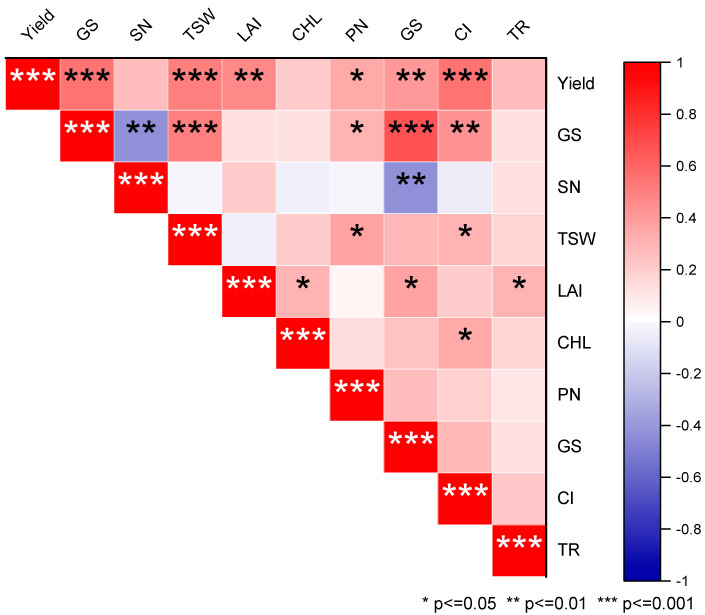
Relationship between yield and other studied parameters. Red represents positive correlation, and blue represents negative correlation (* *p* ≤ 0.05, ** *p* ≤ 0.01, *** *p* ≤ 0.001). The intensity of color represents the significance of a variable. The different abbreviations refer to grains spike^−1^ (GS), spike number (SN), 1000 seed weight (TSW), yield, leaf-area index (LAI), chlorophyll content (Chl), photosynthesis rate (PN), stomatal conductance (GS), intercellular CO_2_ concentration (CI) and transpiration rate (TR), respectively.

According to the Pearson’s correlation analysis, a strong positive correlation showed between yield and intercellular, stomatal conductance, and photosynthesis rates and the 1000-seed index (Figure 7), while, a negative relationship was exhibited by spike number and stomatal conductance. In addition, only a small positive correlation was shown between yield and photosynthesis rate.

## 4. Discussion

Soil is essential for plant survival, nutritional flow, water, and air between soil and crops, which influences plant growth and yield, and, in turn, determines the development of yield and its components [24]. Since the amount of nitrogen fertilizer applied is strongly linked to higher production of crops, farmers strive for a high yield by applying large amounts of fertilizers, resulting in excess nitrogen being released into the air, water, and soil, causing a cascade of human health and environmental issues [25]. The contribution of soil base fertility and nitrogen fertilizer to nutrient application in agriculture is determined, and the soil-nutrient application, abilities, and characteristics are different under different soil fertility, resulting in various attributes of nutrient absorption and utilization by plants, which directly affect the consumption of crop nutrients [26,27]. The highest yield percentage of winter wheat below 200 kg N ha^−1^ is greater in comparison with the other doses. Proper arrangements promote the yield composition of both (nitrogen and cultivars) growth factors, to achieve higher yields than ever before [28,29].

However, Ranva et al. [30] revealed that after a huge quantity of fertilizer, the spike numbers, grains spike^−1^, and 1000-grain weight significantly increased and finally achieved higher grain-yield production. The effect of nitrogen fertilizer on the tiller number in all growth phases of the winter-wheat population varies according to the cultivar differences. To achieve rational control of the tiller number in all the growth phases of the winter-wheat population, the growth and development characteristics of different cultivars, as well as the appropriate amount of nitrogen-fertilizer treatment on the topdressing time, are chosen. Nitrogen application aided the growth of wheat spikes, resulting in an increase in the number of effective spikes and grain yield in spike^−1^ [31,32]. With an increase in nitrogen supply, the nitrogen and the cultivar variables of the winter-wheat yield improved, particularly the 1000-grain weight and grain-number spike^−1^, which reached significant levels. Grain-number spike^−1^ could not reach a significant level when the nitrogen-fertilizer quantity exceeded 100 kg N hm^−1^. However, the 1000-grain weight was significantly increased when the nitrogen-fertilizer quantity exceeded 200–250 kg N hm^−1^, our results were in-line with the Tabak et al. [28]. 

With an increase in the nitrogen supply, all the factors of crop production and yield traits would not show a particular increase in tendency but exhibited the first increase and then reduction in tendency under the nitrogen application of 100 kg N hm^−2^, however, the 1000-grain weight and spike reached the highest at 200 kg N hm^−2^. The number of spikes and yield were highest and the results were significant. The outcomes of this study presented that with the increase in nitrogen application, the spike numbers, grains spike^−1^, 1000-grain weight, and the grain yield first increase and then decrease. The results of this study revealed that with the increase in nitrogen application, the number of spikes, grains spike^−1^ and 1000-grain weight for seeds and the yield increased first and then it decreased, the spike number and yield variance were significant however, the variance among the number of spike plant^−1^ and the 1000-grain weight for seeds was not significant under the 200 kg N ha^−1^ in both cultivars. The number of spike plot^−1^ and yield improved significantly, and there was no significant difference among the number of spikes and the 1000-grain weight, which exhibited that on the basis of both cultivars affecting the yield production of Zhongmai 175, which was because of a stronger nitrogen-utilization capacity that was more reliable with the earlier studies [33,34,35].

The leaf-area size determines the quantity of light capture, which influences the photosynthesis and accumulation of photosynthetic [36]. In this research, it originates with the increase in the 300 kg ha^−1^ treatment (225 kg nitrogen and 75 kg manure) range, that LAI first reduces and then improves. LAI under 300 kg ha^−1^ treatment (225 kg nitrogen and 75 kg manure) is greater at the booting stage for the Zhongmai 175 cultivar as compared to Jindong 22. Below nitrogen application, crops can improve the efficiency of light interception by improve the canopy size, such as improving the leaf-area index to receive more light [37]. Appropriate nitrogen application can increase the single leaf area and the population of the leaf-area index in winter wheat [38], and delaying the nitrogen fertilizer is helpful to postpone the decreased population of leaf area in the later development period of winter wheat [39]. In this research, below the application of the 300 kg ha^−1^ treatment, the 300 kg ha^−1^ treatment (225 kg nitrogen and 75 kg manure) is favorable to improving the LAI of winter wheat. However, improving the LAI of winter wheat requires only a minimal supply of nitrogen. It designates that under nitrogen and manure application, the adaptable influence of nitrogen on leaf area is decreased, and the higher the light degree is, the lesser the effect [40].

The improvement in the photosynthetic pigments might also help capture and use light more efficiently [41,42]. In this research, under nitrogen and manure application, the total chlorophyll improved significantly at the tillering, jointing, and booting stages under the 200 kg ha^−1^ treatment for the Jindong 22 cultivar, and at the heading and flowering stage the highest results were perceived under the 300 kg ha^−1^ treatment. After supplying the nitrogen and manure application, the chlorophyll content of winter wheat improved significantly, and under 100 kg ha^−1^ and 300 kg ha^−1^ (225 kg nitrogen and 75 kg manure) the chlorophyll reduced at the tillering stage in both cultivars. This is because the application of manure helps the regulating synthesis of chlorophyll content, which makes up for the absence of light by increasing the chlorophyll content in the basic metabolism [43,44]. When nitrogen and manure are applied, the Jindong 22 cultivar is improved, and the total chlorophyll is higher than that of the Zhongmai 175 cultivar. Winter-wheat leaves improve the absorption of blue violet light by improving the comparative content of chlorophyll, thus improving the utilization of the small amount of light for crops in weak light [45,46]. Moreover, the chloroplast’s capacity to reduce chemical compounds is improved, and the content of light-collecting developments of green plants and the movement of the photosynthetic organic method are better [47]. Under moderate and unnecessary application environments, the reduction in chlorophyll level might be connected to the absence of light concentration and blockage of chlorophyll synthesis [48,49]. In this research, appropriate nitrogen and manure application is suitable to improving the chlorophyll level of winter wheat without covering under both cultivars. Under moderate and unnecessary application, improving the nitrogen and manure is not favorable to the chlorophyll synthesis. Under nitrogen and manure application, the influence of improving nitrogen fertilizer on chlorophyll synthesis is less than that of common light. This is due to the reduction in light intensity and inadequate absorption power, for shaded leaves of winter wheat, which bounds the photosynthetic carbon absorption and the reasons for complaints about the carbon and nitrogen metabolism, which influence the buildup and transport of photosynthetic components, such as chlorophyll in plants [50,51].

The physical characteristics of plants includes photosynthetic rate, stomatal conductance, transpiration rate, inter-cellular CO_2_ concentrations, and chlorophyll content. Nitrogen is an essential nutrient and a fundamental component of protein synthesis. It is also a basic component of grains and is directly linked to the life activities of plants. As an outcome, nitrogen has been supplied expansively in agricultural produces over the previous few periods to improve winter-wheat-grain yields. Although specific studies have exposed that under the circumstance of higher nitrogen application the photosynthetic rate of leaves was reduced by the application of a huge quantity of nitrogen fertilizer in agricultural production, the harm of fertilizers was directly affected, the soil pollution was sympathetic, and the agricultural environmental reached a malicious round [52,53,54,55].

Zhang et al. [56] exhibited that the light transference of the high amount of nitrogen for winter-wheat production was significantly superior to that of the lesser amount of nitrogen, which might significantly regulate the net photosynthetic traits of the flag leaves of winter wheat. This research exhibited that the application of 200 kg N ha^−1^ enhanced the photosynthetic traits of flag leaves in winter wheat, significantly improving the net photosynthetic rate, stomatal conductance, and transpiration rate of winter wheat after flowers set and significantly reducing the intercellular CO_2_ concentration. This could be because of the high rate of nitrogen application, as well as the different varieties, which enhanced the light sites for production in the agricultural field; while, at the same time, the adequate nitrogen supply made photo-thermal resources more efficient. The nutrient form was appropriate, and the individual plants were well-developed: the green-leaf area was big, the function period was extended, and the aged period of all the plants in the later stage of winter wheat was postponed, so as to avoid the initial maturation of the leaves [39,48,57]. This research exhibited that the photosynthetic activity was closely connected to the crops’ yield, and the leaf-photosynthetic rate was significant purpose for higher crop yields, although nitrogen fertilizer might improve a crops’ capacity to manufacture chlorophyll, so it was one of the greatest active factors to adjust crops-leaf-photosynthetic capability [58,59,60]. 

This research practice describes that after the soil, air, and water content were similar, the photosynthetic rate of winter wheat usually improved first and then became stable; with the improvement of nitrogen application and intercellular CO_2_ concentration, the transpiration rate and net photosynthetic rate normally improved first and then reduced, which similarly designated that an adequate nitrogen supply is suitable for increasing the photosynthesis rate of winter-wheat leaves throughout the flowering stage. Suitable nitrogen application is a feasible way to increase the above-ground photosynthesis rate of winter wheat, improve the development and transfer of dry-matter production, and promote the growth and yield of the plants. The outcomes of this research exhibited that the photosynthetic traits in the winter-wheat leaves of the Zhongmai 175 cultivar with nitrogen 200 kg ha^−1^ and 300 kg ha^−1^ (proportion of 225 kg nitrogen and 75 kg manure) fertilizers all extended the optimum level, which might be due to the suitable nitrogen-fertilizer quantity promoting the chlorophyll synthesis. In addition, protective-enzyme activity in the winter-wheat plants was simultaneously maintained. However, few studies have also revealed that improved nitrogen fertilizer is favorable to enhance the production of winter wheat and improve the canopy atmosphere. However, an unnecessary leaf area will lead to an irrational canopy structure following winter-wheat grain-yield loss [61,62,63,64].

## 5. Conclusions

In conclusion, the nitrogen rates of 200 kg ha^−1^ and 300 kg ha^−1^ (proportion of nitrogen 225 and manure 75 kg ha^−1^) efficiently prompted the leaf-area index, chlorophyll content, photosynthesis rate, intercellular, and yield components of winter wheat at the jointing, heading, and flowering stages. Single application of 200 kg N ha^−1^ and the integrative use of nitrogen 225 and 75 kg ha^−1^ manures promoted the growth and yield traits of the Zhongmai 175 cultivar as compared to Jindong 22 at the jointing and flowering stages. Grain yield was significantly correlated with most of the parameters. Therefore, this study recommended that the combined application of 125 and 225 kg ha^−1^ nitrogen fertilizer along with 75 kg ha^−1^ organic manure is crucial to achieve higher grain yields of winter wheat. In addition, the combination of nitrogen and manure application under different growth stages could be valuable in improving the grain yield of winter wheat.

## Figures and Tables

**Table 1:** Yield and yield traits as affected by the integrative effects of the nitrogen and manure application in winter wheat.

Jindong 22					Zhongmai 175			
Treatments	Grains Spike^−1^	Spike m^−2^	1000 Seed Weight	Yield (kg/ha)	Grains Spike^−1^	Spike m^−2^	1000-Grain Weight	Yield (kg/h)
T1	29.67 d	532.00 d	31.62 d	5720.05 d	34.78 d	453.67 d	31.20 d	5674.05 d
T2	31.11 d	641.00 a	34.79 c	6644.86 c	38.78 c	483.67 a	35.30 d	6692.57 b
T3	42.22 a	656.67 a	38.12 a	8248.19 a	51.33 a	462.67 b	39.27 a	8696.93 a
T4	40.67 a	604.33 c	33.40 b	7912.03 b	48.69 b	481.67 a	36.26 c	8485.25 a
T5	35.11 c	552.67 c	33.41 b	6728.61 c	47.44 b	482.00 a	36.63 b	8416.42 a
T6	37.78 c	636.67 b	33.40 b	7850.29 b	50.33 a	483.33	35.61 b	6662.93 c
T7	39.67 b	573.67 c	35.49 b	8074.86 a	50.00 a	356.67 c	36.24 c	6457.85 c

T1 (control plot), T2 (nitrogen 100 kg^−1^ ha), T3 (N 200 kg^−1^ ha), T4 (N 300 kg^−1^ ha), T5 100 kg^−1^ ha (nitrogen 75% + manure 25%), T6 200 kg^−1^ ha (nitrogen 50% + manure 50%), and T7 300 kg^−1^ ha (nitrogen 25% + manure 75%). Mean values in separate columns followed by similar letters are not significantly different at *p* < 0.05. The values are the mean (*n* = 3).

## Data Availability

Not applicable.
